# Lower Urinary Tract System Symptoms and Urinary Incontinence in Hypertensive Disorders of Pregnancy; A Prospective Observational Comparative Study

**DOI:** 10.3390/jcm15114162

**Published:** 2026-05-28

**Authors:** Mehmet Kagitci, Senol Senturk, Mehmet Kivrak, Savas Ozdemir, Gizem Tetik, Hakki Uzun

**Affiliations:** 1 Department of Obstetrics and Gynecology, Faculty of Medicine, Recep Tayyip Erdogan University, 53100 Rize, Turkey; mehmet.kagitci@erdogan.edu.tr (M.K.); senol.senturk@erdogan.edu.tr (S.S.); savas.ozdemir@erdogan.edu.tr (S.O.); 2 Department of Biostatstics and Medical Informatics, Faculty of Medicine, Recep Tayyip Erdogan University, 53100 Rize, Turkey; 3 Department of Obstetrics and Gynecology, High Specialization Training and Research Hospital, 16000 Bursa, Turkey; gizemkalcioglu@gmail.com; 4 Department of Urology, Faculty of Medicine, Recep Tayyip Erdogan University, 53100 Rize, Turkey; hakki.uzun@erdogan.edu.tr

**Keywords:** hypertensive disorders of pregnancy, urinary incontinence, lower urinary tract symptoms, quality of life, pregnancy

## Abstract

**Background/Objectives**: The aim of this study was to evaluate the association between hypertensive disorders of pregnancy and the frequency of urinary incontinence and lower urinary tract symptoms and to assess the impact of these symptoms on quality of life in pregnant women. **Methods**: This observational comparative study was conducted between March 2024 and September 2025 and included 182 pregnant women between 24 and 40 weeks of gestation. The study group consisted of 91 pregnant women diagnosed with hypertensive disorders of pregnancy, while 91 normotensive pregnant women served as controls. Demographic and obstetric characteristics were recorded. Urinary incontinence and selected lower urinary tract symptoms, as well as the impact of urinary symptoms on quality of life, were assessed using the International Consultation on Incontinence Questionnaire–Short Form, Urinary Distress Inventory-6, and Incontinence Impact Questionnaire-7. Logistic regression analyses were performed to identify independent factors associated with the presence of urinary incontinence. **Results**: Urinary incontinence was significantly more frequent in the hypertensive group compared with controls (65.9% vs. 20.9%, *p* < 0.001). Lower urinary tract symptoms were also more prevalent among hypertensive pregnant women (71.5% vs. 53.8%, *p* = 0.011). UDI-6, ICIQ-SF, and total IIQ-7 scores were significantly higher in the hypertensive group, indicating greater symptom severity and worse quality of life (all *p* < 0.001). In multivariable logistic regression analysis including the entire study population, hypertensive pregnancy was independently associated with urinary incontinence (OR: 8.33, 95% CI: 4.00–16.67, *p* < 0.001), whereas age, body mass index, smoking status, and gravida were not independently associated with UI. **Conclusions**: Hypertensive disorders of pregnancy are strongly and independently associated with an increased frequency of urinary incontinence and lower urinary tract symptoms, as well as a significant deterioration in quality of life. These findings highlight the importance of routine evaluation of urinary symptoms in hypertensive pregnancies and support a multidisciplinary approach to their management.

## 1. Introduction

Hypertensive disorders of pregnancy are common diseases that increase fetal, maternal, and perinatal mortality and morbidity. They occur in approximately 5–10% of all pregnancies, and this rate is increasing [1]. Systemic vascular dysfunction, endothelial damage, and hemodynamic changes are observed in hypertensive pregnancies. Conditions with high blood pressure during pregnancy may be associated with an increased risk of cardiovascular and renal diseases [2].

Urinary incontinence (UI) and lower urinary tract symptoms (LUTSs) are common in pregnancy, and these symptoms can reduce the quality of life and affect the quality of obstetric care throughout pregnancy. Although UI does not completely prevent daily activities in pregnant women, it is a common problem that reduces the quality of life [3]. The frequency of UI in pregnant women has been reported to be between 9% and 75%, and it increases as the gestational week progresses [4]. The frequency of UI has also been found to be higher in non-pregnant women with hypertension than in healthy controls [5]. This situation suggests that hypertension may have a potential impact on bladder function and continence mechanisms.

LUTSs are commonly observed in pregnant women. They are particularly common in the later stages of pregnancy and negatively affect quality of life [6]. Reasons for this include hormonal changes, increased intra-abdominal pressure, and mechanical load on the pelvic floor. A multicenter study reported that 97.3% of pregnant women had at least one lower urinary tract symptom [7]. The frequency of hypertensive disorders, LUTSs, and UI is reported to be increased in pregnant women. To our knowledge, no studies have specifically investigated the independent association between hypertensive disorders of pregnancy and lower urinary tract symptoms or urinary incontinence.

This study aimed to evaluate the effect of hypertensive disorders of pregnancy on the frequency of urinary incontinence and lower urinary tract symptoms and the impact of these symptoms on the quality of life of pregnant women.

## 2. Materials and Methods

This prospective observational comparative study was conducted at the Department of Obstetrics and Gynecology, Recep Tayyip Erdogan University Training and Research Hospital, between 1 March 2024 and 7 September 2025. Pregnant women between 24 and 40 weeks of gestation were included in the study. The participants’ age, gravida, previous delivery method, gestational age, body mass index, smoking status, education, and occupation information were recorded. Smoking status was defined as active cigarette smoking during pregnancy. Passive smoking exposure and smoking history before pregnancy were not evaluated in the present study. The study group consisted of patients with hypertensive disorders of pregnancy, and the control group consisted of patients with normal blood pressure values. The diagnosis of hypertensive disorders of pregnancy was made as follows: Normotensive: Pregnant women with blood pressure values systolic < 140 mmHg and diastolic < 90 mmHg were considered normotensive. Gestational Hypertension: Patients with blood pressure readings above 140/90 mmHg on two measurements taken 4 h apart, first detected after 20 weeks of gestation, without proteinuria or target organ involvement. Chronic Hypertension: Hypertension diagnosed before pregnancy or first diagnosed before 20 weeks of gestation. Preeclampsia: Blood pressure readings above 140/90 mmHg on two measurements taken 4 h apart, or above 160/110 mmHg on a single measurement, first detected after 20 weeks of gestation, in addition to the presence of proteinuria. In patients without proteinuria; platelet count < 100,000, renal failure (serum creatinine > 1.1 or doubling of serum creatinine), hepatic dysfunction (doubling of ALT and AST values), pulmonary edema, or new onset headache unresponsive to drug treatment. In addition to demographic and obstetric characteristics, laboratory parameters including alanine aminotransferase (ALT), aspartate aminotransferase (AST), platelet count, and hemoglobin levels were recorded to better characterize the hypertensive pregnancy population and evaluate systemic involvement associated with hypertensive disorders of pregnancy.

Participants’ urinary incontinence status, urinary symptoms, and the impact of urinary symptoms on their quality of life were investigated. For this purpose, the “International Consultation on Incontinence Questionnaire—Short Form” (ICIQ-SF), Urinary Distress Inventory-6 (UDI-6), and the Incontinence Impact Questionnaire-7 (IIQ-7) were used. ICIQ-SF: This is a form that assesses the frequency of urinary incontinence and its impact on quality of life. A validated Turkish version was used [8]. Higher scores indicate greater severity of urinary incontinence and its impact on quality of life. UDI-6: This is a form that assesses the frequency and severity of urinary incontinence and urinary symptoms. Higher scores indicate more severe symptoms. A validated Turkish version was used. IIQ-7: This is a form that assesses the impact of urinary incontinence on quality of life. In this form, travel, physical activity, social relationships, and emotional health can be evaluated separately. A validated Turkish form was used [9]. Lower urinary tract symptoms were evaluated based on the subjective complaints of the participants in accordance with the criteria defined by the International Continence Society (ICS). Frequency was defined as the individual’s perception of urinating more frequently than normal during the day; nocturia was defined as waking up during the main sleep period to go to the toilet to urinate [10,11].

Inclusion criteria: Pregnant women aged 18–49 years and between 24–40 weeks of gestation were included in the study. Exclusion criteria: Pregnant women with diabetes, gestational diabetes, neurological disease, multiple pregnancies, urinary tract infection, or a history of urinary incontinence before pregnancy were excluded from the study. Additionally, patients diagnosed with severe preeclampsia were excluded for ethical reasons. The sample size for this study was calculated using the G*Power program (Version 3.1.9.7). At 90% statistical power and α = 0.05 significance level, the minimum required sample size was calculated as 182 (Control: 91—Study: 91) according to the independent proportions test [12].

Statistical Analysis: All statistical analyses were performed using IBM SPSS Statistics (version 25) and Jamovi (version 2.4.6). Continuous variables were first evaluated for normality using the Shapiro–Wilk test. Normally distributed variables were presented as mean ± standard deviation (SD) and compared using the independent samples Student’s *t*-test. Non-normally distributed variables were presented as median (minimum–maximum) and compared using the Mann–Whitney U test. Categorical variables were summarized as number (percentage) and compared using the chi-square test or Fisher’s exact test when appropriate. Effect sizes were reported to complement *p*-values. Cohen’s d was calculated for normally distributed continuous variables. For non-normally distributed variables, effect size (r) was calculated using the formula r = Z/√N and interpreted as small (0.10), moderate (0.30), or large (0.50). For categorical variables, Cramer’s V was calculated and interpreted as small (0.10), moderate (0.30), and large (0.50). For urinary symptom severity and quality-of-life scores (IIQ-7, ICIQ-SF, and UDI-6), group comparisons were performed using the Mann–Whitney U test due to non-normal distribution. Median (minimum–maximum) values and corresponding effect sizes (r) were reported. To identify independent factors associated with urinary incontinence (UI), logistic regression analyses were performed. Univariable logistic regression was first conducted for each variable. Variables of clinical relevance (age, body mass index, smoking status, and gravida) were included in the multivariable logistic regression model irrespective of univariable significance. Odds ratios (ORs) with 95% confidence intervals (CIs) were reported. Model fit was assessed using −2 log likelihood, Cox & Snell R^2^, and Nagelkerke R^2^ statistics. Model calibration was evaluated with the Hosmer–Lemeshow goodness-of-fit test. Linearity of the logit for continuous variables was examined using the Box–Tidwell procedure. Multicollinearity was assessed using variance inflation factors (VIF), with values < 2 indicating no evidence of collinearity. Influential observations were evaluated using standardized residuals and Cook’s distance. All statistical tests were two-tailed, and a *p*-value < 0.05 was considered statistically significant.

## 3. Results

A total of 182 pregnant women were included in the study, 91 in the hypertension group and 91 in the control group. The demographic data of the participants included in the study are shown in Table 1.

**Figure 1:**
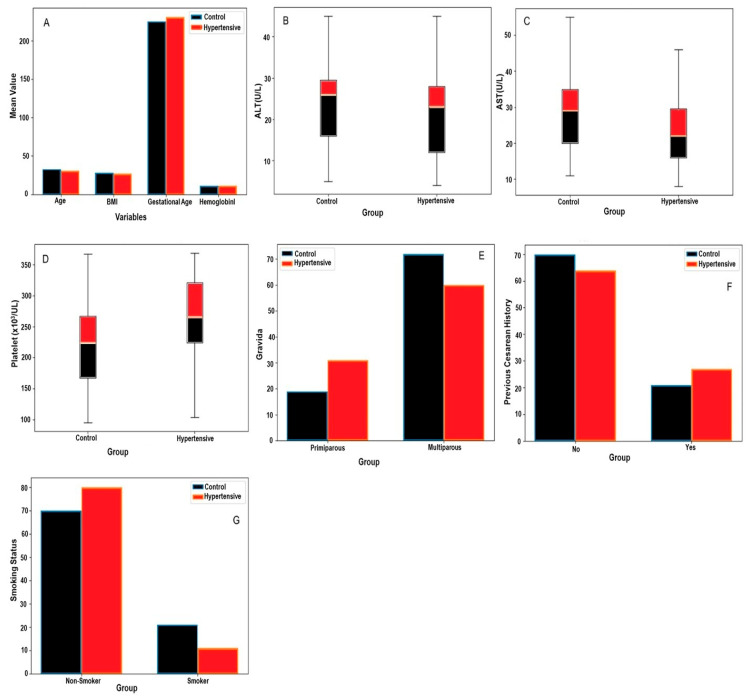
Characteristics of normotensive and hypertensive pregnant women. (**A**) Mean values of age, body mass index (BMI), gestational age, and hemoglobin levels. (**B**–**D**) Boxplot distributions of alanine aminotransferase (ALT), aspartate aminotransferase (AST), and platelet counts. (**E**–**G**) Distribution of parity, previous cesarean section history, and smoking status between groups.

As shown in Table 1, women with hypertensive disorders of pregnancy were significantly older and had higher body mass index (BMI) compared to normotensive controls (age: *p* = 0.025; BMI: *p* = 0.003). Gestational age did not differ significantly between the groups (*p* = 0.119). Liver enzymes demonstrated significant differences, with both alanine aminotransferase (ALT) (*p* = 0.007) and aspartate aminotransferase (AST) (*p* < 0.001) levels differing between groups. Platelet count also showed a statistically significant difference (*p* < 0.001), with a small-to-moderate effect size (r = 0.23). Hemoglobin levels were comparable between groups (*p* = 0.471). Regarding obstetric characteristics, parity distribution differed significantly (*p* = 0.034), and smoking was more frequent in the hypertensive group (*p* = 0.039). No significant difference was observed in previous cesarean section history (*p* = 0.20) (Figure 1).

According to Table 2, Education level did not differ significantly between hypertensive and normotensive groups (*p* = 0.576), with a negligible effect size (Cramer’s V = 0.08). Occupation distribution showed a statistically significant difference (*p* = 0.020), although the effect size was small-to-moderate (V = 0.19). Symptom presence was significantly more frequent among hypertensive women (*p* = 0.022), with a small-to-moderate association (V = 0.17). The strongest association was observed for urinary incontinence type (*p* < 0.001), demonstrating a large effect size (V = 0.46), indicating a markedly different distribution of incontinence subtypes between the two groups.

**Figure 2:**
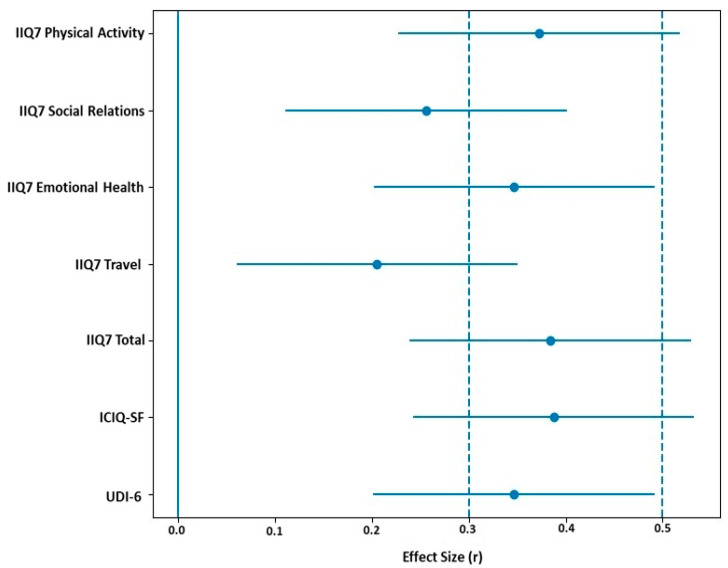
Forest plot of symptom score effect sizes. Points represent effect sizes (r) calculated from Mann–Whitney U tests comparing groups. Horizontal lines indicate 95% confidence intervals. The solid vertical line at r = 0 represents no effect. Dashed vertical lines indicate thresholds for moderate (r = 0.30) and large (r = 0.50) effect sizes. Higher effect size values reflect greater clinical differences between groups.

As presented in Table 3, hypertensive pregnant women demonstrated significantly higher urinary symptom severity and quality-of-life scores compared to normotensive controls. All IIQ-7 subdomains (physical activity, social relations, emotional health, and travel) were significantly different between groups (all *p* < 0.001), with small-to-moderate effect sizes (r = 0.32–0.43). The total IIQ-7 score showed a large effect size (r = 0.62), indicating a substantial difference in quality-of-life impact between groups. Similarly, both ICIQ-SF and UDI-6 total scores were significantly higher in the hypertensive group (*p* < 0.001), with large (r = 0.65) and moderate-to-large (r = 0.54) effect sizes, respectively (Figure 2). Exploratory subgroup analyses according to hypertensive disorder subtype are presented in Supplementary Table S1. Preeclampsia cases generally demonstrated higher urinary symptom scores and urinary incontinence frequency compared with the other hypertensive subgroups; however, these findings should be interpreted cautiously due to the limited sample size of the gestational and chronic hypertension groups.

In the univariable logistic regression analysis, hypertensive pregnancy was significantly associated with urinary incontinence. Compared with normotensive women, hypertensive pregnant women had substantially higher odds of urinary incontinence (OR = 7.35, 95% CI: 3.70–14.29, *p* < 0.001). In the multivariable model adjusted for age, BMI, smoking status, and gravida, hypertensive pregnancy remained independently associated with urinary incontinence (adjusted OR = 8.33, 95% CI: 4.00–16.67, *p* < 0.001). None of the covariates age, BMI, smoking status, or gravida were independently associated with urinary incontinence (all *p* > 0.05) (Table 4).

## 4. Discussion

The data we obtained in this study showed that the frequency of UI and LUTSs is significantly higher in hypertensive pregnant women compared to normotensive pregnant women. It also shows that HDP patients are more affected by the decrease in quality of life due to UI and urinary symptoms compared to non-HDP pregnant women. Advanced maternal age and increased BMI are higher in HDP patients. Hypertension has also been identified as a strong risk factor for UI.

In the first trimester of pregnancy, vasodilation occurs due to the effects of estrogen and progesterone. This leads to a tendency for low systemic blood pressure. As pregnancy progresses, especially after 20 weeks, sympathetic system and baroreceptor sensitivity increase. Kidney dilation develops. Glomerular filtration rate increases. Excessive sympathetic activity during this period may contribute to the development of preeclampsia and hypertension. In the third trimester, blood pressure begins to rise again towards baseline levels [1]. All these physiological mechanisms that enable adaptation to pregnancy may also be contributing factors to hypertensive diseases seen in pregnant women.

LUTSs are quite common in pregnant women. In one study, the frequency of LUTSs in pregnant women was reported as 99.2% [13]. In another study, the frequency of UI in pregnant women was determined as 52%, showing a significant decrease in quality of life [14]. In our study, the frequency of UI and LUTSs in hypertensive pregnant women was found to be 65.9% and 71.5%, respectively. The frequency of UI and LUTSs was found to be significantly higher compared to normotensive pregnant women. Logistic regression analysis showed that the presence of hypertension is a strong independent risk factor for UI. Our results are consistent with the literature. Furthermore, it shows that hypertensive pregnant women have a higher risk for UI and LUTSs. In exploratory subgroup analyses, preeclampsia cases tended to demonstrate higher urinary symptom scores and urinary incontinence frequency compared with gestational and chronic hypertension subgroups. However, these findings should be interpreted cautiously because the number of patients in the gestational and chronic hypertension groups was limited.

The negative impact of LUTSs and UI on quality of life during pregnancy has been well documented [14,15]. Both decreased physical function and limitations in social and emotional areas have been reported, with many pregnant women experiencing difficulties in daily living activities due to these symptoms. In our study, the impact on quality of life was found to be even higher in hypertensive pregnant women. In this study, it was observed that pregnant women with hypertension were significantly older and had a higher body mass index compared to normotensive controls. Both advanced maternal age and increased body mass index have previously been identified as significant risk factors for urinary incontinence and lower urinary tract symptoms during pregnancy. In parallel, UDI-6 and ICIQ-SF scores were significantly higher in the hypertensive group, showing more severe urinary symptoms and a greater negative impact on quality of life. However, in multivariate logistic regression analysis, age and body mass index were not found to be independently associated with urinary incontinence; this suggests that hypertensive pregnancy itself may be a dominant risk factor that overrides the contribution of traditional demographic variables.

The potential effects of hypertensive disorders of pregnancy on urinary incontinence and lower urinary tract symptoms can be explained by multifactorial mechanisms. It is thought that the systemic endothelial dysfunction, increased oxidative stress, and inflammatory response, which are prominent in hypertensive pregnancies, may negatively impact bladder vascularization and detrusor function [16]. Disruption of the endothelium-derived vasodilator vasoconstrictor balance can lead to decreased bladder perfusion and increased afferent nerve activity, triggering storage symptoms such as urgency, frequency, and nocturia. Furthermore, increased sympathetic nervous system activity associated with hypertension has been found to be associated with increased detrusor overactivity and bladder sensitivity [17]. The combination of increased intra-abdominal pressure and mechanical stress on the pelvic floor during pregnancy, along with the accompanying vascular and neuromuscular changes in hypertensive pregnancies, may lead to earlier and more pronounced impairment of continence mechanisms. These pathophysiological processes constitute the possible biological basis that could explain the identification of hypertensive pregnancy as a strong risk factor for urinary incontinence, independent of age and body mass index, in our study.

In the present study, hypertensive pregnant women demonstrated significantly higher UDI-6 and ICIQ-SF scores compared with normotensive controls, indicating both increased severity of lower urinary tract symptoms and a greater negative impact on quality of life. These findings may be mechanically explained by the pathophysiological changes associated with hypertensive disorders of pregnancy. Consistent with these mechanisms, hypertensive pregnant women in our study demonstrated significantly higher UDI-6 and ICIQ-SF scores, indicating increased severity of storage symptoms and greater impairment in quality of life. These findings suggest that the clinical burden observed in hypertensive pregnancies reflects the functional consequences of the pathophysiological processes outlined above. The combined effect of these vascular, neurogenic, and mechanical pathways provides a plausible explanation for the substantially worse symptom burden and quality-of-life impairment observed in hypertensive pregnant women in this study.

Strengths of the present study include its prospective design, the use of validated and widely accepted questionnaires (UDI-6 and ICIQ-SF), and the inclusion of a well-defined hypertensive pregnancy cohort. The use of multivariable logistic regression analysis allowed adjustment for key confounders such as age, body mass index, smoking status, and gravida. However, several limitations should be acknowledged. First, although exploratory subgroup analyses according to hypertensive disorder subtype were performed, the limited sample sizes within the gestational and chronic hypertension groups restricted definitive comparative interpretation. Second, lower urinary tract symptoms and urinary incontinence were assessed using validated self-reported questionnaires without objective urodynamic or physiological confirmation. Third, although multivariable adjustment was performed for relevant confounders, residual confounding due to unmeasured variables cannot be entirely excluded. Furthermore, while the study was prospectively conducted, the analytical design reflects an observational comparison, and therefore causal inferences between hypertensive pregnancy and urinary incontinence cannot be established. Finally, as the study was conducted in a single-center population, the generalizability of the findings to broader and more diverse obstetric populations may be limited.

## 5. Conclusions

In conclusion, hypertensive disorders of pregnancy are independently associated with a markedly increased risk of urinary incontinence and a greater burden of lower urinary tract symptoms, resulting in significant impairment in quality of life. These findings suggest that urinary symptoms may represent an underrecognized clinical component of hypertensive pregnancies. Routine screening for UI and LUTSs during obstetric follow-up may facilitate earlier recognition and multidisciplinary management, ultimately improving maternal well-being.

## Figures and Tables

**Table 1:** Baseline characteristics of the study and control groups. Data are presented as mean ± standard deviation for normally distributed variables, median (minimum–maximum) for non-normally distributed variables, and number (percentage) for categorical variables. Comparisons between groups were performed using Student’s *t*-test for normally distributed continuous variables and Mann–Whitney U test for non-normally distributed variables. Categorical variables were compared using the fisher exact chi-square test. Effect sizes are presented as Cohen’s d for normally distributed variables (*) and rank-biserial correlation (r) for non-normally distributed variables (**). A *p*-value < 0.05 was considered statistically significant. **BMI**: body mass index; **ALT**: alanine aminotransferase; **AST**: aspartate aminotransferase. Bold values indicate statistical significance.

Variables		Control Group	Study Group	Total	Effect Size (Cohen’s d */r **)	*p*-Value
Age (years)		30.66 ± 4.65	32.37 ± 5.53	31.52 ± 5.17	0.33 *	**0.025**
BMI (kg/m^2^)		27.21 ± 2.32	28.44 ± 3.13	27.83 ± 2.81	0.44 *	**0.003**
Gestational Age (days)		231.04 ± 28.2	224.86 ± 24.93	227.95 ± 26.72	0.23 *	0.119
ALT (U/L)		26 (5–98)	23 (4–45)	25 (4–98)	0.41 **	**0.007**
AST (U/L)		29 (11–112)	22 (8–66)	25.5 (8–112)	0.49 **	**<0.001**
Platelet (×10^3^/µL)		224 (95–367)	265 (104–369)	247 (95–369)	0.23 **	**<0.001**
Hemoglobin (g/dL)		11.4 ± 1.46	11.26 ± 1.25	11.34 ± 1.36	0.1 *	0.471
Gravida	Primiparous	31 (34.1)	19 (20.9)	50 (27.5)	-	**0.034**
	Multiparous	60 (65.9)	72 (79.1)	132 (72.5)	-	
Previous Cesarean History	Yes	27 (29.7)	21 (23.1)	48 (26.4)	-	0.2
	No	64 (70.3)	70 (76.9)	134 (73.6)	-	
Smoking Status	Smoker	11 (12.1)	21 (23.1)	32 (17.6)	-	**0.039**
	Non Smoker	80 (87.9)	70 (76.9)	150 (82.4)	-	

**Table 2:** Comparison of sociodemographic and urinary symptoms characteristics. Data are presented as number (percentage). Associations between categorical variables and study groups were evaluated using the chi-square test. Effect sizes are reported as Cramer’s V and interpreted as small (0.10), moderate (0.30), and large (0.50). Bold values indicate statistical significance. A *p*-value < 0.05 was considered statistically significant.

Variable	Category	Control n (%)	Hypertensive n (%)	Total n (%)	*p*-Value	Effect Size (Cramer’s V)
Education	Primary School	17 (18.7)	13 (14.3)	30 (16.5)	0.576	0.08
	Middle School	10 (11.0)	14 (15.4)	24 (13.2)		
	High School	36 (39.6)	31 (34.1)	67 (36.8)		
	University	28 (30.8)	33 (36.3)	61 (33.5)		
Occupation	Housewife	51 (56.0)	61 (67.0)	112 (61.5)	**0.020**	0.19
	Healthcare/Education	30 (33.0)	21 (23.1)	51 (28.0)		
	Other	10 (11.0)	9 (9.9)	19 (10.4)		
Symptom Presence	Asymptomatic	42 (46.2)	26 (28.6)	68 (37.4)	**0.022**	0.17
	Symptomatic	49 (53.8)	65 (71.4)	114 (62.6)		
Urinary Incontinence Type	None	72 (79.1)	31 (34.1)	103 (56.6)	**<0.001**	0.46
	Stress	9 (9.9)	33 (36.3)	42 (23.1)		
	Urge	4 (4.4)	15 (16.5)	19 (10.4)		
	Mixed	6 (6.6)	12 (13.2)	18 (9.9)		

**Table 3:** Comparison of urinary symptom scores. Data are presented as median (min–max). Between-group comparisons were performed using the Mann–Whitney U test. Effect size was calculated as r (Z/√N) and interpreted as small (0.10), moderate (0.30), and large (0.50). Higher scores indicate greater symptom severity and poorer quality of life. A *p*-value < 0.05 was considered statistically significant. Bold values indicate statistical significance.

Variable	Control Median (Min–Max)	Hypertensive Median (Min–Max)	Effect Size (r)	*p*-Value
IIQ-7 Physical Activity	0 (0–2)	2 (0–4)	0.41	**<0.001**
IIQ-7 Social Relations	0 (0–0)	0 (0–1)	0.32	**<0.001**
IIQ-7 Emotional Health	0 (0–1)	1 (0–3)	0.43	**<0.001**
IIQ-7 Travel	0 (0–0)	0 (0–1)	0.38	**<0.001**
IIQ-7 Total	0 (0–4)	5 (2–9)	0.62	**<0.001**
ICIQ-SF	0 (0–5)	6 (3–11)	0.65	**<0.001**
UDI-6	0 (0–3)	3 (1–6)	0.54	**<0.001**

**Table 4:** Logistic regression analysis for urinary incontinence. Logistic regression analyses were performed to evaluate factors associated with urinary incontinence (UI). The outcome variable was urinary incontinence (UI = 1). Odds ratios (OR) with 95% confidence intervals (CI) are presented for univariable and multivariable models. Normotensive pregnancy was used as the reference category. For the univariable model including hypertensive pregnancy status only, model fit statistics were as follows: Cox & Snell R^2^ = 0.24 and Nagelkerke R^2^ = 0.32. For the multivariable model adjusted for age, body mass index (BMI), smoking status, and gravida, Cox & Snell R^2^ was 0.29 and Nagelkerke R^2^ was 0.39. Model calibration was assessed using the Hosmer–Lemeshow goodness-of-fit test (*p* > 0.05 indicating adequate fit). Linearity of the logit for continuous variables was evaluated using the Box–Tidwell procedure. Multicollinearity was assessed using variance inflation factors (VIF), with all values < 2 indicating no evidence of collinearity. Statistical significance was defined as *p* < 0.05. Bold values indicate statistical significance.

Variable	N	Univariable OR (95% CI)	*p*-Value	Multivariable OR (95% CI)	*p*-Value
Normotensive	91	1 (reference)	–	1 (reference)	–
Hypertensive pregnancy (yes vs. no)	91	7.35 (3.70–14.29)	<0.001	8.33 (4.00–16.67)	**<0.001**
Age (per year)	182	1.02 (0.96–1.08)	0.49	0.99 (0.93–1.06)	0.87
BMI (kg/m^2^)	182	1.03 (0.93–1.14)	0.56	0.97 (0.86–1.09)	0.62
Smoking (yes vs. no)	182	0.78 (0.35–1.73)	0.55	0.79 (0.33–1.91)	0.6
Gravida (per unit)	182	0.95 (0.71–1.27)	0.73	0.90 (0.63–1.28)	0.55

## Data Availability

The datasets used and/or analyzed during the current study are available from the corresponding author on reasonable request.

## Supplementary Materials

The following supporting information can be downloaded at https://www.mdpi.com/article/10.3390/jcm15114162/s1, Table S1: Exploratory subgroup analysis according to hypertensive disorder subtype.

## Abbreviations

The following abbreviations are used in this manuscript:

HDP	Hypertensive disorders of pregnancy
UI	Urinary incontinence
LUTS	Lower urinary tract symptom
ICIQ-SF	International Consultation on Incontinence Questionnaire–Short Form
UDI-6	Urinary Distress Inventory-6
IIQ-7	Incontinence Impact Questionnaire-7
ALT	Alanine aminotransferase
AST	Aspartate aminotransferase
OR	Odds Ratio
