# Supplementary material for: Lower Urinary Tract System Symptoms and Urinary Incontinence in Hypertensive Disorders of Pregnancy; A Prospective Observational Comparative Study
Source: J Clin Med. 2026 May 28;15(11):4162. doi: 10.3390/jcm15114162 (PMC13257796; doi:10.3390/jcm15114162)
Supplement: Supplementary file 1 [file jcm-15-04162-s001.zip › jcm-4259220-supplementary.pdf]

Supplementary Table S1. Exploratory subgroup analysis according to hypertensive disorder subtype. Data are presented descriptively due to the limited sample size within certain subgroups. Categorical variables were compared using the Fisher's exact test, and continuous variables were compared using the Kruskal–Wallis test. Due to the very small sample sizes in the gestational and chronic hypertension subgroups, these exploratory results should be interpreted cautiously. ICIQ-SF, International Consultation on Incontinence Questionnaire–Short Form; UDI-6, Urinary Distress Inventory-6; IIQ-7, Incontinence Impact Questionnaire-7.

Variable	Preeclampsia (n=84)	Gestational Hypertension (n=4)	Chronic Hypertension (n=3)	p-value
Urinary incontinence, n (%)	59 (70.2)	1 (25.0)	0 (0.0)	**0.009**
Lower urinary tract symptoms, n (%)	60 (71.4)	3 (75.0)	2 (66.7)	0.971
ICIQ-SF, median (min–max)	6 (0–19)	0 (0–9)	0 (0–0)	**0.048**
UDI-6, median (min–max)	4 (0–12)	0.5 (0–11)	0 (0–0)	**0.027**
IIQ-7 Total, median (min–max)	5 (0–17)	0 (0–14)	0 (0–0)	0.067
